# Incidence, Demographics, and Service Utilization of Patients Referred for Renal Replacement Therapy at the Instituto Mexicano del Seguro Social (IMSS) Michoacán: A Retrospective Analysis

**DOI:** 10.7759/cureus.80579

**Published:** 2025-03-14

**Authors:** Christian Diaz de Leon Castañeda, Venice Chávez Valencia, Cleto Álvarez Aguilar, Virginia Robinson Fuentes

**Affiliations:** 1 Researchers for Mexico (Investigadoras e Investigadores por México) Program, Secretaría de Ciencia, Humanidades, Tecnología e Innovación (SECIHTI), Mexico City, MEX; 2 Faculty of Nursing, Universidad Michoacana de San Nicolás de Hidalgo, Morelia, MEX; 3 Department of Nephrology, Hospital General Regional No. 1, Instituto Mexicano del Seguro Social (IMSS), Morelia, MEX; 4 Faculty of Medical and Biological Sciences "Dr. Ignacio Chávez", Universidad Michoacana de San Nicolás de Hidalgo, Morelia, MEX

**Keywords:** chronic kidney disease (ckd), hemodialysis services, peritoneal dialysis (pd), renal replacement therapy (rrt), social security

## Abstract

Background: Chronic kidney disease (CKD) is a health condition with high tangible and intangible implications for patients, health systems, and society in Mexico. This study aims to analyze the incidence, demographics, and service utilization of patients referred for renal replacement therapy (RRT) involving peritoneal dialysis (PD) or hemodialysis (HD) in Care units of the Instituto Mexicano del Seguro Social (IMSS) in the state of Michoacán, Mexico.

Methods: A database from IMSS Michoacán containing information from January 2007 to September 2023 was analyzed. Special analyses were performed by subdelegations of adscription of the patients and according to a "region of interest" comprising three municipalities in eastern Michoacán (Zinapecuaro, Hidalgo, and Maravatío). This analysis included incidence rates of patient referrals, mean age at dialysis initiation, and percentages of service provision.

Results: An increasing trend in the incidence of cases referred for RRT was identified. In 2021, the state incidence rate of new cases referred for RRT was 28.42 cases per 100,000 population. However, this rate was markedly higher in the subdelegation of Zitácuaro and the region of interest. The analysis of patients' demographic characteristics showed that the majority were male (n=1494; 60.6%), with a global mean age at dialysis initiation of 50.3 (±17.00 SD) years. However, patients from the subdelegation of Zitácuaro and the region of interest were identified to be significantly younger than those from other municipalities. The analysis of RRT service provision showed a trend over time towards a higher proportion of HD services, with some inequalities in access to HD services.

Conclusions: There is an increasing trend in RRT referrals at IMSS Michoacán. A more significant issue in the incidence of RRT referrals and the mean age at dialysis initiation was identified for a particular subdelegation of adscription and region of interest of the IMSS Michoacán. Moreover, the need to strengthen HD service infrastructure is identified to improve accessibility for insured patients living in various municipalities in Michoacán, Mexico.

## Introduction

Chronic kidney disease (CKD) is a condition that progressively impairs kidney function. The etiology of CKD includes intrinsic causes such as genetic factors, sex, and age, and extrinsic causes such as poor lifestyle, diet quality, chronic diseases including diabetes and hypertension, exposure to exogenous substances including medications, consumption of alcohol and tobacco, drugs of abuse, industrial food additives, and exposure to environmental agents including heavy metals, pesticides, and chemicals encountered during occupational exposure [1].

CKD can progress to end-stage renal disease (ESRD), leading to multiple complications for the patient, including the accumulation of toxins in the body, which causes damage to various organs and tissues, and impaired electrolyte and mineral excretion, which can increase blood pressure and lead to its associated complications. Renal damage can manifest directly through histological changes in renal biopsy or indirectly through albuminuria, abnormalities in urine sediment, or imaging techniques; however, serum creatinine or C cystatin are often the most common biomarkers detected in primary care [2]. Various international guidelines, including the 2024 Kidney Disease Improving Global Outcomes (KDIGO) guidelines, have confirmed the definition of CKD (regardless of clinical diagnosis) as the presence of an estimated glomerular filtration rate (eGFR) below 60 ml/minute/1.73 m² and/or kidney damage for at least three months [3].

Creatinine clearance is often used to calculate the glomerular filtration rate (GFR) to determine the progression of CKD. Five stages of CKD progression have been proposed based on GFR levels: Stage 1 with normal or high GFR (GFR > 90 mL/minute), Stage 2 mild CKD (GFR = 60-89 mL/minute), Stage 3A moderate CKD (GFR = 45-59 mL/minute), Stage 3B moderate CKD (GFR = 30-44 mL/minute), Stage 4 severe CKD (GFR = 15-29 mL/minute), and Stage 5 end-stage CKD (GFR < 15 mL/minute) [3]. The latter is characterized by the need for renal replacement therapy (RRT), either through peritoneal dialysis (PD), hemodialysis (HD), or kidney transplantation. The treatment of CKD patients is interdisciplinary, as it is essential to manage various aspects of the patient's care such as diet, physical activity, avoiding risk factors, and controlling chronic diseases if present.

In countries undergoing demographic and epidemiological transition, the aging population and the high prevalence of metabolic diseases such as type 2 diabetes (T2D) and arterial hypertension (AHT) also lead to a high prevalence of CKD, particularly in the adult population [4]. Moreover, CKD carries significant economic implications for the patient, healthcare services, and society, with the economic burden increasing as the disease progresses. Early CKD onset leads to lost years of productive life, years of life lost due to premature death, and more years lived with disability.

In addition to the genetic and metabolic diseases mentioned, the presence of behavioral risk factors such as an obesogenic lifestyle and substance abuse (alcohol, tobacco, or drugs), as well as exposure to environmental factors, can exacerbate the problem, leading to earlier onset of CKD and potentially faster progression in individuals with these risk factors. Therefore, it is necessary to monitor the incidence and prevalence of the disease nationally and regionally, as well as the potential behavioral and environmental risk factors involved.

In Mexico, demographic and epidemiological transitions have led to a high prevalence of non-communicable chronic diseases (NCDs). The international Global Burden of Disease (GBD) project has highlighted the CKD problem in Mexico. A growing epidemiological trend has been identified, and by 2019, Mexico ranked third in Latin America for mortality and disability-adjusted life years (DALYs) lost due to CKD [5]. Additionally, in 2020, a national prevalence of 9.29% was determined, including causes such as T2D (1.42%), type 1 diabetes (0.05%), hypertension (0.41%), glomerulonephritis (0.19%), and other unspecified causes (7.22%) [6]. It is worth noting that idiopathic CKD has been frequently classified in Mexico [7-10].

A recent publication estimated the average annual per capita cost of RRT for social security institutions in Mexico, including the Instituto Mexicano del Seguro Social (IMSS) and the Instituto de Seguridad y Servicios Sociales de los Trabajadores del Estado (ISSSTE), both of which serve the formal employed population in the private and public sectors. In the first year of treatment, the annual dialysis, hemodialysis, and kidney transplantation costs were 465,500, 783,800, and 799,400 Mexican pesos (23,900, 40,300, and 41,100 USD, considering an exchange rate of 1 Mexican peso = 0.05141 USD), respectively [11].

Several publications have explored the CKD problem in Mexico to varying degrees [12,13]. The GBD study has also allowed for a regional understanding of the problem by state, based on prevalence, incidence, or disease burden indicators such as years of life lost due to premature death (YLL), where it was determined that Veracruz, Tabasco, State of Mexico, Mexico City, Tlaxcala, and Puebla topped the list in 2021 [14]. Additionally, more specific analyses have been conducted for particular regions of the country where high incidence rates have been identified, potentially associated with various factors such as environmental, social, socio-economic, cultural, biological, or genetic factors, with a focus on idiopathic CKD and research on incidence and risk factors in children, adolescents, and young adults. Some of these regional studies have been conducted in Veracruz, Jalisco, Aguascalientes, and San Luis Potosí from different approaches [15-20]. Moreover, other regions of the country have been identified where high mortality rates among children and adolescents due to CKD have been reported, but more extensive analyses have not been published [21]. Recently, there has been growing interest in research in certain municipalities in the eastern region of Michoacán, mainly because a high incidence of CKD has been observed in young people without metabolic diseases; however, research on adults remains important. A recent study explored the prevalence of CKD among IMSS beneficiaries in the state of Michoacán; however, it only explored metabolic risk factors and did not examine differences in prevalence or incidence by geographic region [22].

This study aims to analyze the incidence, demographics, and service utilization among IMSS beneficiaries in Michoacán who are referred for RRT involving PD or HD. Specifically, the main research hypotheses are: (i) Incidence of patient referrals to RRT might be higher in certain IMSS Michoacán subdelegations of adscription or regions, (ii) Age at dialysis initiation might be lower in certain IMSS Michoacán subdelegations of adscription or regions, and (iii) Provision of dialysis services might be different in the IMSS Michoacán subdelegations of adscription.

## Materials and methods

This was a study conducted at Universidad Michoacana de San Nicolás de Hidalgo (UMSNH), Morelia, Mexico, based on data from a database created by the IMSS Michoacán. The study was approved and agreed upon via a collaboration agreement between UMSNH and IMSS, signed in July 2024 (registration number: UMSNH/DVSS/63/2004).

According to the latest national census conducted in 2020, the population of the state of Michoacán was 4,748,846 [23]. The state of Michoacán is comprised of 113 municipalities. The IMSS is the social security system accessible to workers with formal employment. According to the 2020 national census, the IMSS covered 1,207,045 inhabitants in Michoacán, representing 25.42% of the population [23]. In Michoacán, IMSS services at the primary care level are provided through five subdelegations: Morelia, Uruapan, Zamora, Zitácuaro, and Lázaro Cárdenas, each with their respective primary care services named Unidades de Medicina Familiar (UMF) [24]. Additionally, at the second and third levels of healthcare, the IMSS in Michoacán has different kinds of service provision schemes: Regional General Hospital (HGR), General Zone Hospital (HGZ), General Subzone Hospital (HGS), General Zone Hospital with Family Medicine (HGZMF), General Subzone Hospital with Family Medicine (HGSMF), Ambulatory Care Medical Unit (UMAA), and UMF with Hospitalization (UMFH) [25].

**Figure 1:**
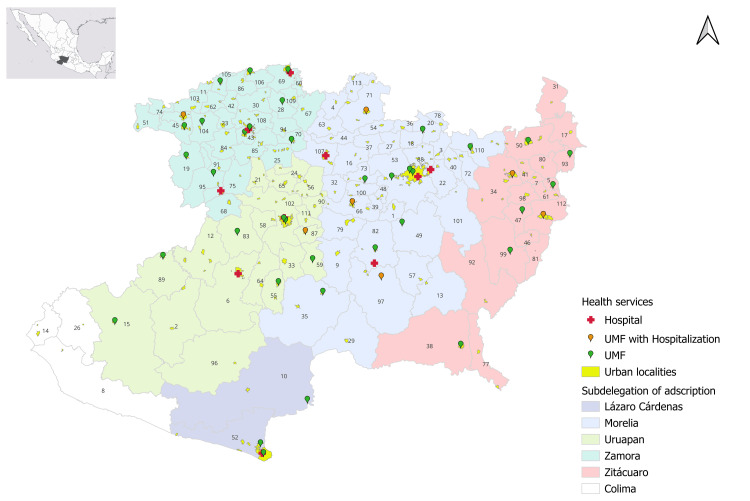
Health services at IMSS Michoacán, Mexico. Municipalities: 1. Acuitzio, 2. Aguililla, 3. Álvaro Obregón, 4. Angamacutiro, 5. Angangueo, 6. Apatzingán, 7. Aporo, 8. Aquila, 9. Ario, 10. Arteaga, 11. Briseñas, 12. Buenavista, 13. Carácuaro, 14. Coahuayana, 15. Coalcomán de VázquezPallares, 16. Coeneo, 17. Contepec, 18. Copándaro, 19. Cotija, 20. Cuitzeo, 21. Charapan, 22. Charo, 23. Chavinda, 24. Cherán, 25. Chilchota, 26. Chinicuila, 27. Chucándiro, 28. Churintzio, 29. Churumuco, 30. Ecuandureo, 31. Epitacio Huerta, 32. Erongarícuaro, 33. Gabriel Zamora, 34. Hidalgo, 35. La Huacana, 36. Huandacareo, 37. Huaniqueo, 38. Huetamo, 39. Huiramba, 40. Indaparapeo, 41. Irimbo, 42. Ixtlan, 43. Jacona, 44. Jiménez, 45. Jiquilpan, 46. Juárez, 47. Jungapeo, 48. Lagunillas, 49. Madero, 50. Maravatío, 51. Marcos Castellanos, 52. Lázaro Cárdenas, 53. Morelia, 54. Morelos, 55. Múgica, 56. Nahuatzen, 57. Nocupétaro, 58. Nuevo Parangaricutiro, 59. Nuevo Urecho, 60. Numarán, 61. Ocampo, 62. Pajacuarán, 63. Panindícuaro, 64. Parácuaro, 65. Paracho, 66. Pátzcuaro, 67. Penjamillo, 68. Peribán, 69. La Piedad, 70. Purépero, 71. Puruándiro, 72. Querándaro, 73. Quiroga, 74. Cojumatlán de Regules, 75. LosReyes, 76. Sahuayo, 77. San Lucas, 78. Santa Ana Maya, 79. Salvador Escalante, 80. Senguio, 81. Susupuato, 82. Tacámbaro, 83. Tancítaro, 84. Tangamandapio, 85. Tangancácuaro, 86. Tanhuato, 87. Taretán, 88. Tarímbaro, 89. Tepalcatepec, 90. Tingambato, 91. Tinguindín, 92. Tiquicheo de Nicolás Romero, 93. Tlalpujahua, 94. Tlazazalca, 95. Tocumbo, 96. Tumbiscatío, 97. Turicato, 98. Tuxpan, 99. Tuzantla, 100. Tzintzuntzan, 101. Tzitzio, 102. Uruapan, 103. Venustiano Carranza, 104. Villamar, 105. Vista Hermosa, 106. Yurécuaro, 107. Zacapu, 108. Zamora, 109. Zináparo, 110. Zinapécuaro, 111. Ziracuaretiro, 112. Zitácuaro, 113. José Sixto Verduzco IMSS: Instituto Mexicano del Seguro Social

A map was created to show the location of the IMSS subdelegations in Michoacán and the location of health services. The location of health services was obtained from the database "Catalog of Unique Health Establishment Codes-CLUES" for 2023 [26]. The open-source program QGIS ver. 3.34 was used [27]. Figure 1 shows the generated map.

Database of CKD patients

A database of IMSS beneficiaries in Michoacán who were referred to peritoneal dialysis and hemodialysis services was obtained. This database was generated by implementing an electronic patient registration system. The data to be analyzed was taken from January 2007 to September 2023.

Data analysis

The database was analyzed using IBM SPSS Statistics for Windows, Version 25.0 (Released 2017; IBM Corp., Armonk, New York, United States). All the patients registered in the database were included in the analysis because all were referred to RRT services, regardless of the diagnosis registered in the database (no inclusion/exclusion criteria for patients were used). Regarding the data cleaning process, duplicate registers of patients were eliminated using patients' personal identification numbers, so only the first register was used. No imputation techniques were employed, and the descriptive analysis of particular variables was performed only on the patients with information about that variable.

First, a trend analysis was conducted on the number of new cases recorded in the database over time. General trend graphs and descriptive tables were obtained. An analysis by sex, age group (<30 years and >30 years), and the patient's subdelegation of adscription, i.e., the administration to which the UMF where they receive care belongs (primary care level), was included. A cut-off of 30 years was proposed to establish two age groups, as it is considered unlikely that chronic non-communicable diseases (CNCDs) of metabolic origin (diabetes, hypertension) would be determined as the cause below this age.

The second analysis conducted was a description of the causes recorded in the database, for which pie charts were created for the total number of patients entered into the database, as well as for those under 30 years of age and over 30 years of age. The frequency of cases recorded with CNCDs of metabolic origin, such as type 1 diabetes (T1D), T2D, and arterial hypertension (AHT), as well as those recorded simply as "Chronic Kidney Disease," was analyzed. A table was also created to describe the causes recorded in the database.

The third analysis was a calculation of the incidence rates of patients referred to PD/HD services in 2021 since recent information about population and IMSS affiliations were available from the previous census conducted in 2020 [23]. These incidence rates were calculated by sex, age group, Subdelegation of adscription of the patients, and a region of interest composed of three municipalities in the state of Michoacán (Zinapécuaro, Maravatío, and Hidalgo).

The fourth analysis involved calculating the age at dialysis initiation as an indicator that may reflect the presence or exposure to different risks that may influence the development and progression of CKD to the need for RRT. Descriptive graphs such as histograms and boxplots were created, as well as descriptive tables including the analysis of the influence of sex and the geographical location of patients, which was obtained through the subdelegation of adscription to which the UMF they attended belonged. This analysis was performed for the total number of patients entered into the database. The tables reported the frequency of patients in two age categories (<30 years and >30 years). This analysis was also performed by the Care unit that attended to the patients. Additionally, an analysis was conducted to compare the age at dialysis initiation in municipalities in the eastern part of Michoacán with other municipalities in the state. Differences in means (DM) were calculated to compare these groups.

Finally, the absolute and relative frequency of patients who were in different types of service was obtained: automated peritoneal dialysis, continuous ambulatory peritoneal dialysis, in-house hemodialysis, and outsourced hemodialysis, analyzing by the Care unit attended by the patients and by the subdelegation of adscription to which the primary care clinic to which they are affiliated belonged.

Descriptive statistics was employed in all the analyses performed since data came from the total RRT-referred patients insured to the IMSS Michoacán in the study period. The approach of the study was to describe the data available.

## Results

The database included 2,526 patients, of which 2,484 (98.3%) were still active in the IMSS healthcare services when downloaded (September 2023). This means they had not been removed for various reasons (such as changing institutions, migration, loss of eligibility, transplantation, or death). Of the patients included, 1,531 (60.6%) were men, and 995 (39.4%) were women. Information on the age at dialysis initiation was available for 2,471 patients, the overall mean age at dialysis initiation was 50.30 (±16.97 SD) years, 417 were under 30 years old (16.8%), and 2,064 (83.2%) were over 30 years of age. A more detailed analysis will be presented in a later section.

Trends in patient referrals

**Figure 2:**
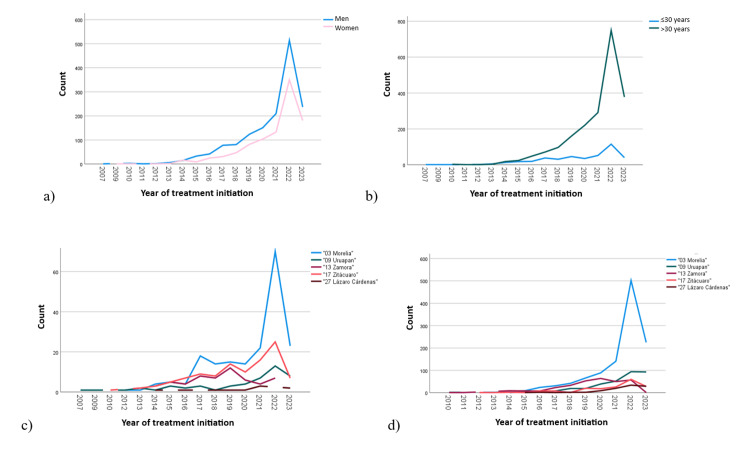
Evolution of patients referrals to RRT services (2007-2023) (a) by sex, (b) by age group, (c) by subdelegation of adscription for patients under 30 years of age, and (d) by subdelegation of adscription for patients over 30 years of age RRT: renal replacement therapy

The trends in the registration of new patients referred for PD and HD are shown in Figure 2. The graphs show a growing trend in registered cases, suggesting an increase in prevalence and incidence each year. Figure 2a illustrates a higher incidence in men and shows a significant increase in cases registered in 2022. Figure 2b indicates a higher incidence of patients over 30 years of age. Figures 2c-2d break down the incidence of new cases by subdelegation of adscription, highlighting the subdelegations of Morelia and Zitácuaro for patients under 30 years of age and the subdelegations of Morelia and Uruapan for those over 30 years of age.

Table 1 describes the incidence of new cases by subdelegation of adscription. In some cases, the year they began operations can be observed due to recent service openings; for example, in the "HGZ 8 Uruapan," which started registering new patients in 2022.

**Table 1:** Evolution of new patient registrations in the database for all causes (2007-2023), by subdelegation of adscription

Variables	2007		2009		2010		2011		2012		2013		2014		2015		2016		2017		2018		2019		2020		2021		2022		2023		TOTAL
Age (years)	< 30	> 30	< 30	> 30	< 30	> 30	< 30	> 30	< 30	> 30	< 30	> 30	< 30	> 30	< 30	> 30	< 30	> 30	< 30	> 30	< 30	> 30	< 30	> 30	< 30	> 30	< 30	> 30	< 30	> 30	< 30	> 30	
Foreigners, n	0	0	0	0	0	0	0	0	0	0	0	0	1	0	0	0	1	0	0	0	0	0	1	0	0	1	0	2	0	2	0	2	10
Morelia, n	0	0	0	0	0	2	0	0	1	0	1	2	4	6	5	9	4	24	18	31	14	42	15	66	14	89	22	141	70	502	23	225	1330
Uruapan, n	1	0	1	0	0	0	0	0	1	0	2	0	1	1	3	5	2	6	3	8	1	19	3	19	4	39	7	52	13	94	8	93	386
Zamora, n	0	0	0	0	0	1	0	1	0	0	0	0	3	9	5	8	4	8	8	23	7	33	12	53	6	64	4	50	7	57	0	1	364
Zitácuaro, n	0	0	0	0	1	0	0	0	0	1	2	1	3	2	5	1	7	8	9	8	8	1	14	20	10	18	16	26	25	60	7	28	281
Lázaro Cárdenas, n	0	0	0	0	0	0	0	0	0	0	0	0	1	0	0	1	1	2	0	1	1	2	1	2	1	9	3	20	0	34	2	29	110
TOTAL	1	0	1	0	1	3	0	1	2	1	5	3	13	18	18	24	19	48	38	71	31	97	46	160	35	220	52	291	115	749	40	378	2481

Analysis of registered causes for patients in the database

**Figure 3:**
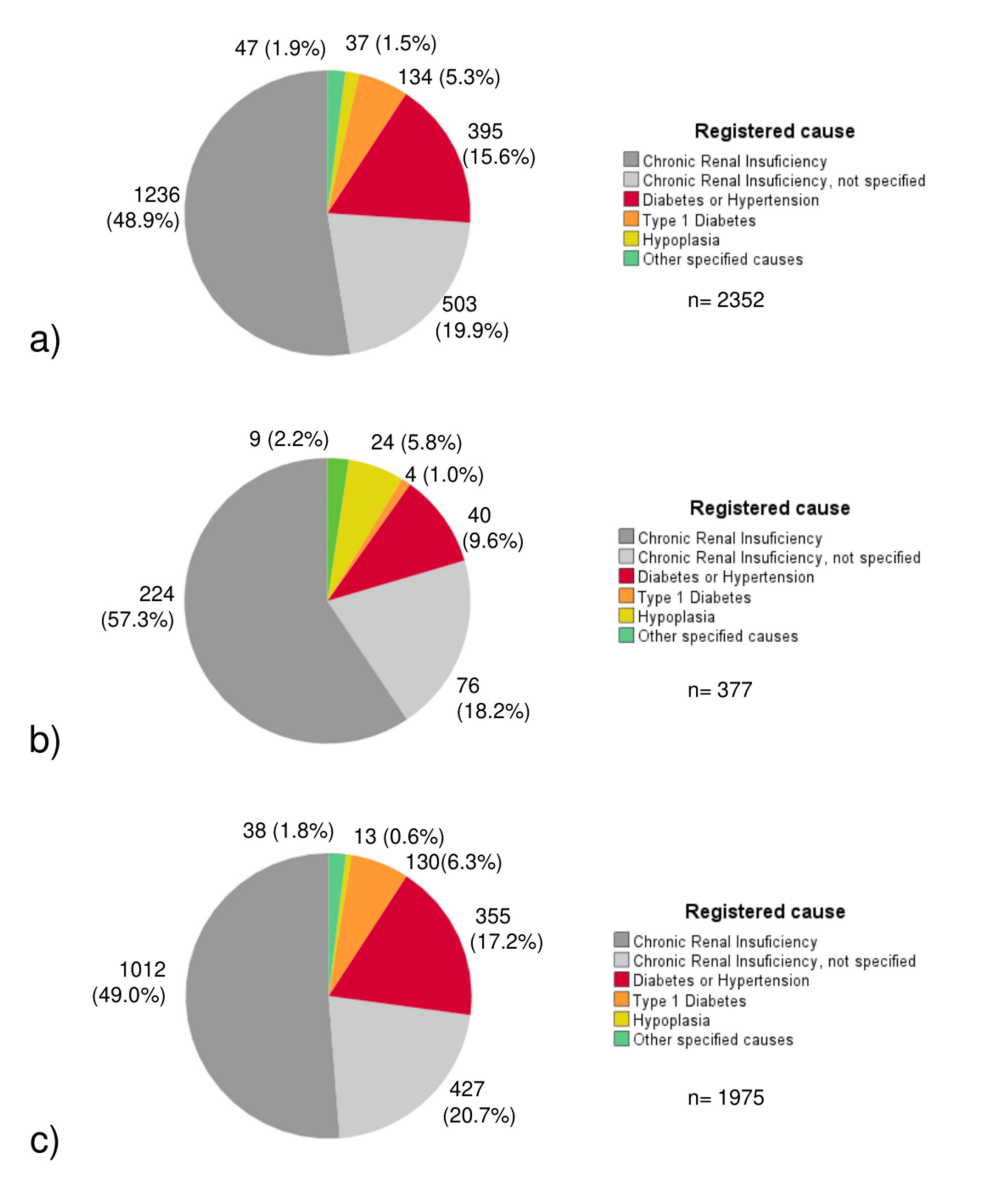
Pie chart of the causes of CKD recorded in the database for the referred patients (2007-2023). (a) Total sample (n=2352), (b) Subjects under 30 years of age (n=377), and (c) Subjects over 30 years of age (n=1975) CKD: chronic kidney disease

Figure 3 shows the frequencies of registered causes of all the referred patients recorded in the database with complete data (n=2352) (Figure 3a), as well as for patients under 30 years of age (n=377, 16.0%) (Figure 3b) and over 30 years of age (n=1,975, 84.0%) (Figure 3c). The majority of causes registered in the database were only as “Chronic Renal Insuficiency" (CRI) or “Chronic Renal Insuficiency, not specified" (CRIns). It stands out that for patients aged under 30 years, there is a major frequency of registration as “CRI” or “CRIns” and hypoplasia, but a minor frequency of causes like “diabetes and hypertension” and “type 1 diabetes”, compared with patients over 30 years. 

Analysis of the incidence of patients referred for PD/HD in 2021

Table 2 shows the incidence of new cases referred to PD/HD by sex, age group, subdelegation of adscription, and region for 2021. The overall rate for this year and the state of Michoacán was 28.42 cases per 100,000 insured persons. Men had a higher incidence rate than women (35.93 and 21.36 cases per 100,000 insured persons, respectively). The incidence rate by age group increased with age, peaking in the 60-69 age group, after which it began to decrease. 

**Table 2:** Analysis of the incidence of new cases referred to dialysis treatment (PD/HD) for all causes (2021), by sex, age group, subdelegation of adscription, and region of interest (N=343) Note: Population and affiliation data are from the 2020 Census (Instituto Nacional de Estadística y Geografía (National Institute of Statistics and Geography) (INEGI), 2020 [23]. PD: peritoneal dialysis; HD: hemodialysis; IMSS: Instituto Mexicano del Seguro Social

Categories		Michoacán population in 2020, n	IMSS Michoacán-affiliated population in 2020, n (%)	New cases admitted in 2021, n	Incidence rate in 2021 (per 100,000 insured individuals)
Sex	Men	2,306,341	584,464 (25.3)	210	35.93
	Women	2,442,505	622,581 (25.5)	133	21.36
Age group (years)	0-9	871,455	187,920 (21.6)	0	0.00
	10-19	834,612	194,596 (23.3)	8	4.11
	20-29	757,999	194,039 (25.6)	43	22.16
	30-39	663,710	179,870 (27.1)	41	22.79
	40-49	569,843	151,597 (26.6)	45	29.68
	50-59	445,998	122,739 (27.5)	76	61.92
	60-69	318,853	96,758 (30.3)	85	87.85
	70-79	181,270	54,073 (29.8)	39	72.12
	≥ 80	101,576	25,439 (25.0)	6	23.59
	Not specified	3,500	14 (0.4)	-	-
Subdelegation of adscription	Non-residents	-	-	2	
	Morelia	1,879,109	560,086 (29.8)	163	29.10
	Uruapan	907,134	202,204 (22.3)	59	29.18
	Zamora	1,016,491	262,478 (25.8)	54	20.57
	Zitácuaro	683,306	74,009 (10.8)	42	56.75
	Lázaro Cárdenas	216,335	105,046 (48.6)	23	21.90
Region of interest	Non-residents	-	-	2	-
	Region of interest	264,028	30,530 (11.6)	49	160.50
	Other municipalities	4,484,818	1,176,515 (26.2)	292	24.82
TOTAL	-	4,748,846	1,207,045 (25.42%)	343	28.42

**Figure 4:**
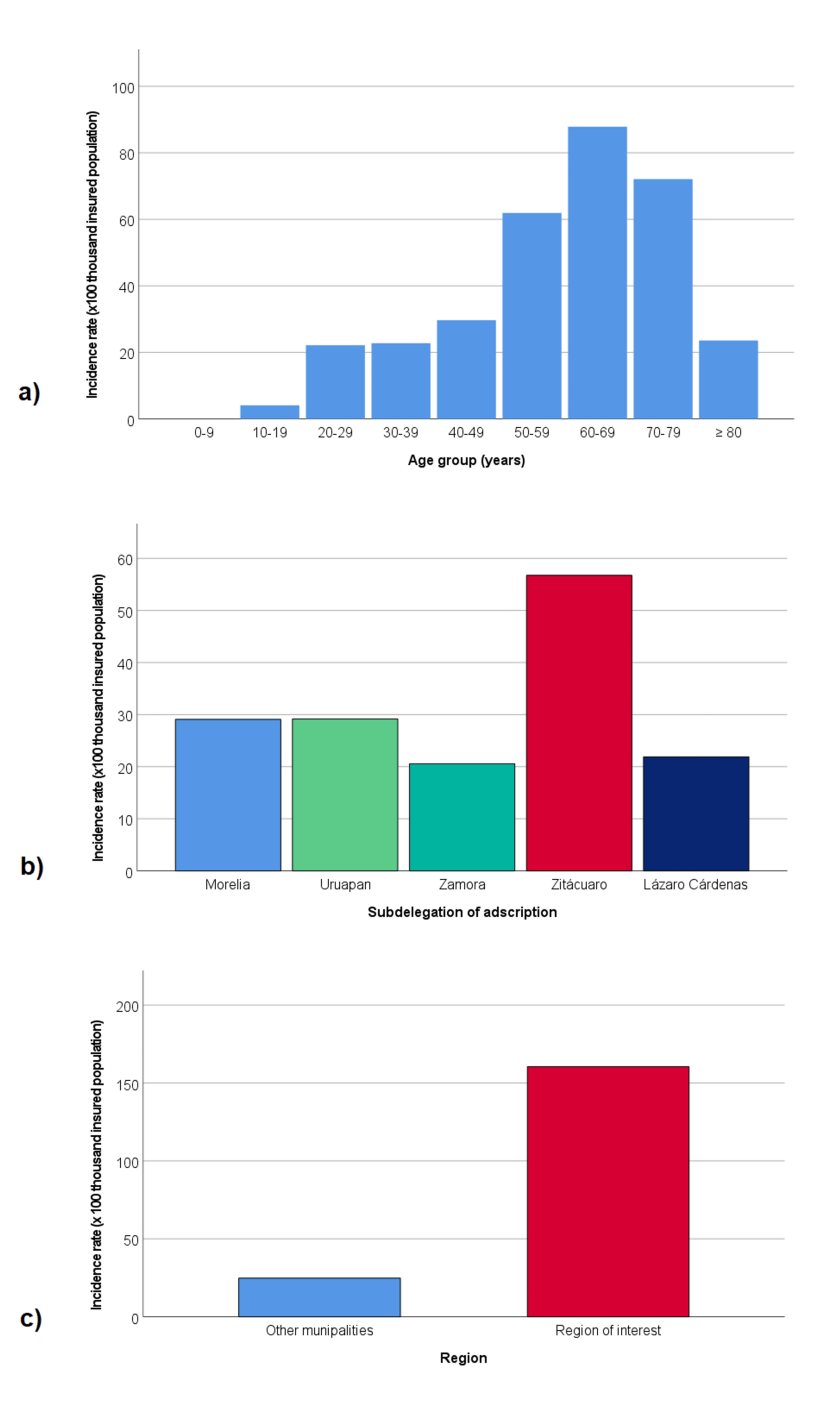
Incidence rate of patient referrals to RRT services (2021) (x 100,000 insured population) (a) by age group, (b) by subdelegation of adscription, and (c) by region RRT: renal replacement therapy

In the analysis of incidence by subdelegation of adscription, the Zitácuaro subdelegation stands out with the highest incidence (56.75 cases per 100,000 insured persons), followed by the Morelia subdelegation (29.10 cases per 100,000 insured persons), Uruapan subdelegation (29.18 cases per 100,000 insured persons), Lázaro Cárdenas subdelegation (21.90 cases per 100,000 insured persons), and Zamora subdelegation (20.57 cases per 100,000 insured persons). The special analysis for the region of interest, which includes the municipalities of Hidalgo, Zinapecuaro, and Maravatio, showed a rate of 160.50 cases per 100,000 insured persons, significantly higher than the rate for the rest of the municipalities in the state of Michoacán, which was 24.82 cases per 100,000 insured persons. Figure 4 shows a graphic representation of the main Incidence rate 2021 results.

Analysis of the age at dialysis initiation

**Figure 5:**
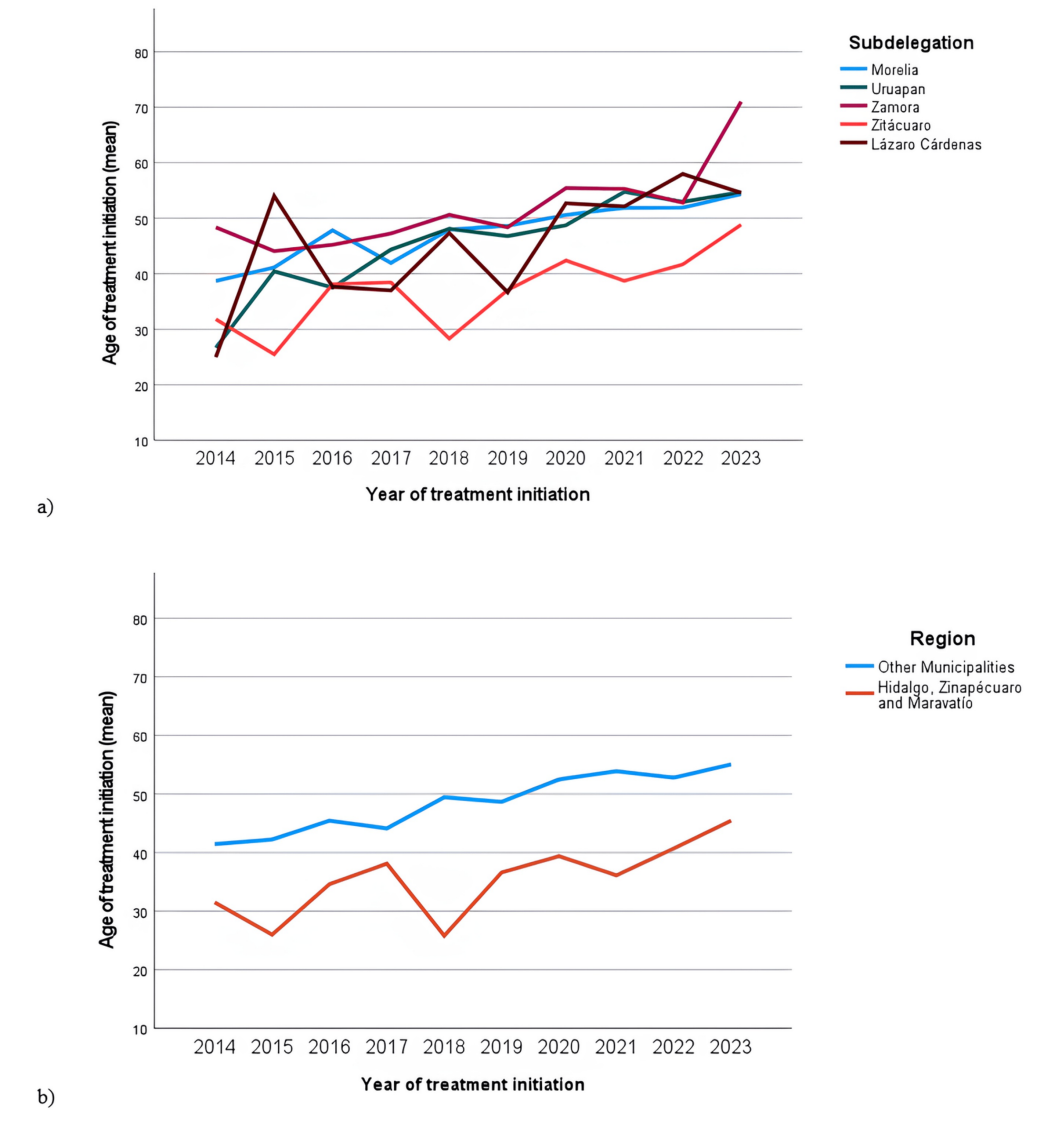
Trends of age at dialysis initiation (mean) (2014-2023) (a) by subdelegation of adscription and (b) by region of interest

Figure 5 summarizes the trends in the age at dialysis initiation (mean) of the patients referred to PD/HD from 2014 to 2023. Table 3 shows the description of all the cases referred to PD/HD and registered in the database (2007-2023) by subdelegation of adscription and region of interest, detailing sex and age at dialysis initiation, as well as the cases of patients under 30 years of age. As mentioned before, information on the age at dialysis initiation was available for 2471 patients, of which 1494 were men (60.5%) and 977 were women (39.5%). The overall mean age at dialysis initiation was 50.30 (±16.97 SD) years. Regarding age groups, 417 were under 30 years of age (16.8%), and 2064 were over 30 years of age (83.2%).

**Table 3:** Demographic characteristics of all patients registered in the database at dialysis initiation for all causes (2007-2023), by subdelegation of adscription (N=2471)

Categories		Total, n	Sex, n (%)				Age at dialysis initiation				Age category at dialysis initiation, n (%)				
			F		M		Mean	SD	Min	Max	≤ 30 years			> 30 years	
			n	%	n	%					n		%	n	%
Subdelegation of adscription	Morelia	1330	538	40.5	792	59.5	51.28	16.86	6.92	92.58	191	14.4		1139	85.6
	Uruapan	386	146	37.8	240	62.2	51.89	16.26	8.00	87.67	50	13.0		336	87.0
	Zamora	364	122	33.5	242	66.5	51.41	16.30	7.08	84.50	56	15.4		308	84.6
	Zitácuaro	281	119	42.3	162	57.7	40.68	17.44	8.92	85.33	107	38.1		174	61.9
	Lázaro Cárdenas	110	52	47.3	58	52.7	53.82	13.57	21.83	84.92	10	9.1		100	90.9
Region of interest	Other municipalities	2143	838	39.1	1305	60.9	51.95	16.38	6.92	92.58	284	13.3		1859	86.7
	Region of interest	328	139	42.4	189	57.6	39.50	16.82	8.92	82.58	130	39.6		198	60.4
TOTAL		2471	977	39.5	1494	60.5	50.30	17.00	6.92	92.58	417	16.8		2064	83.2

**Figure 6:**
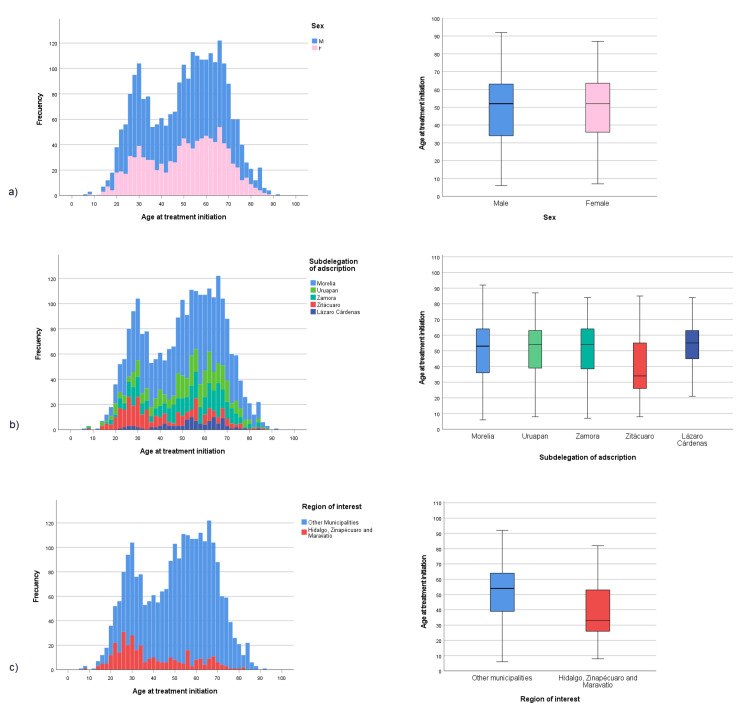
Distribution of age at dialysis initiation for the total number of patients registered (2007-2023) (n=2471) (a) by sex, (b) by subdelegation of adscription, (c) by region of interest

 Figure 6 shows histograms and box plots describing the age at dialysis initiation for patients referred to PD/HD, considering sex, subdelegation of adscription, and region of the state. Age at dialysis initiation histograms showed bimodal patterns.

The age at dialysis initiation by demographic characteristics is graphically shown in Figure 6a. The histograms and box plots showed that the distribution is very similar between men and women. For men, the mean age was 49.61±17.22 years (n=1498), while for women, the mean age was 50.20±16.67 years (n=983), with a mean difference of 0.59 years. This result shows that men, in addition to having a higher incidence, start PD/HD treatment slightly earlier than women.

Regarding the age at dialysis initiation in different subdelegations of adscription, demographic characteristics are shown in Table 4 and graphically in Figure 6b. The mean age for the Morelia subdelegation was 51.28±16.86 years (n=1330), for the Uruapan subdelegation it was 51.89±16.26 years (n=386), for the Zamora subdelegation, the mean age was 51.41±16.30 years (n=364), for Zitácuaro subdelegation, it was 40.68±17.44 years (n=281), and for Lázaro Cárdenas subdelegation, it was 53.82±13.57 years (n=110). The results for the Zitácuaro subdelegation are particularly noteworthy, as patients from this subdelegation started treatment at a younger age compared to other subdelegations. With regard to the region of interest, the mean age at dialysis initiation was 39.50±16.82 (n=328), while for the rest of the municipalities, it was 51.95±16.38 (n=2143), with a mean difference of 12.45 years (Table 3).

**Table 4:** Demographic characteristics of all patients registered in the database at dialysis initiation for all causes (2007-2023), by subdelegation of adscription (N=2471) HGZMF: General Hospital of the Zone with Family Medicine; HGSMF: Subzone General Hospital with Family Medicine; UMF: Family Medicine Unit; UMAA: Ambulatory Medical Care Unit; UMFH: Family Medicine Unit with Hospitalization

Subdelegation	Adscription Unit	Total, n	Sex					Age at dialysis initiation				Age category at dialysis initiation, n (%)					
			F			M		Mean	SD	Min	Max	≤ 30 years			> 30 years		
			n		%	n	%					n		%	n		%
Morelia	HGSMF 24 Pedernales	11	6	54.5		5	45.5	53.55	14.72	28.17	75.42	1	9.1		10	90.9	
	HGZMF 2 Zacapu	74	26	35.1		48	64.9	53.43	15.39	21.17	87.33	8	10.8		66	89.2	
	UMF 3 Quiroga	13	8	61.5		5	38.5	53.55	17.19	25.83	83.17	1	7.7		12	92.3	
	UMF 42 Cuitzeo	30	16	53.3		14	46.7	50.16	16.47	23.33	80.83	5	16.7		25	83.3	
	UMF 43 Churumuco	1	1	100.0		0	0.0	50.58		50.58	50.58	0	0.0		1	100.0	
	UMF 46 La Huacana	3	1	33.3		2	66.7	53.81	19.25	31.58	65.25	0	0.0		3	100.0	
	UMF 65 Villa Madero	12	4	33.3		8	66.7	55.58	10.72	37.92	74.75	0	0.0		12	100.0	
	UMF 70 Zinapécuaro	104	40	38.5		64	61.5	42.29	16.61	16.08	81.08	31	29.8		73	70.2	
	UMF 71 Morelia	5	1	20.0		4	80.0	59.23	16.25	39.33	83.67	0	0.0		5	100.0	
	UMF 74 Tacámbaro	28	13	46.4		15	53.6	49.57	17.32	16.58	79.17	5	17.9		23	82.1	
	UMF 80 Morelia	335	131	39.1		204	60.9	54.88	16.14	6.92	86.50	28	8.4		307	91.6	
	UMF 84 Tacicuaro	112	65	58.0		47	42.0	50.71	14.82	16.58	76.83	12	10.7		100	89.3	
	UMF 85 Tarímbaro	181	68	37.6		113	62.4	47.64	16.32	14.92	78.25	35	19.3		146	80.7	
	UMF 75 -UMAA 254 Morelia	282	103	36.5		179	63.5	52.15	17.61	15.92	92.58	38	13.5		244	86.5	
	UMFH 20 Pátzcuaro	94	38	40.4		56	59.6	55.30	15.81	12.17	84.67	8	8.5		86	91.5	
	UMFH 25 Puruarán	13	4	30.8		9	69.2	46.32	19.52	19.83	75.08	5	38.5		8	61.5	
	UMFH 64 Puruándiro	32	13	40.6		19	59.4	40.69	18.89	20.25	76.33	14	43.8		18	56.3	
	Total	1330	538	40.5		792	59.5	51.28	16.86	6.92	92.58	191	14.4		1139	85.6	
Uruapan	HGSMF 9 Apatzingán	104	43	41.3		61	58.7	50.18	17.43	13.08	84.42	18	17.3		86	82.7	
	UMF 11 Nueva Italia	23	9	39.1		14	60.9	50.13	17.20	19.58	82.42	4	17.4		19	82.6	
	UMF 52 Nuevo Urecho	2	0	0.0		2	100.0	47.83	26.16	29.33	66.33	1	50.0		1	50.0	
	UMF 57 Tancítaro	9	2	22.2		7	77.8	48.40	11.59	23.08	59.42	1	11.1		8	88.9	
	UMF 58 Tepalcatepec	2	0	0.0		2	100.0	72.08	3.42	69.67	74.50	0	0.0		2	100.0	
	UMF 73 Uruapan	2	2	100.0		0	0.0	71.33	0.94	70.67	72.00	0	0.0		2	100.0	
	UMF 76 Uruapan	78	25	32.1		53	67.9	53.32	15.39	19.42	85.00	9	11.5		69	88.5	
	UMF 81 Uruapan	151	62	41.1		89	58.9	52.49	15.52	8.00	87.67	14	9.3		137	90.7	
	UMFH 26 Taretán	15	3	20.0		12	80.0	50.31	19.75	15.75	77.25	3	20.0		12	80.0	
	Total	386	146	37.8		240	62.2	51.89	16.26	8.00	87.67	50	13.0		336	87.0	
Zamora	HGSMF 17 Los Reyes	35	13	37.1		22	62.9	53.97	15.11	22.00	79.50	4	11.4		31	88.6	
	UMF 13 Cotija	7	3	42.9		4	57.1	52.67	17.24	26.33	70.58	1	14.3		6	85.7	
	UMF 21 Jacona	32	10	31.3		22	68.8	53.50	17.54	22.33	83.83	4	12.5		28	87.5	
	UMF 28 Sta. Clara	6	1	16.7		5	83.3	36.04	16.43	22.00	62.75	4	66.7		2	33.3	
	UMF 54 Purépero	3	0	0.0		3	100.0	52.53	22.45	27.75	71.50	1	33.3		2	66.7	
	UMF 6 Jiquilpan	8	4	50.0		4	50.0	48.34	13.32	29.83	65.08	1	12.5		7	87.5	
	UMF 68 Vista Hermosa	5	0	0.0		5	100.0	51.53	20.76	29.08	75.58	1	20.0		4	80.0	
	UMF 72 Yurécuaro	21	5	23.8		16	76.2	42.58	15.02	18.92	83.08	5	23.8		16	76.2	
	UMF 77 La Piedad	95	32	33.7		63	66.3	49.09	16.69	17.17	77.92	19	20.0		76	80.0	
	UMF 82 Zamora	126	45	35.7		81	64.3	54.54	15.44	7.08	84.50	11	8.7		115	91.3	
	UMFH 5 Sahuayo	26	9	34.6		17	65.4	49.75	15.86	18.83	68.17	5	19.2		21	80.8	
	Total	364	122	33.5		242	66.5	51.41	16.30	7.08	84.50	56	15.4		308	84.6	
Zitácuaro	UMF 10 Jungapeo	9	2	22.2		7	77.8	50.36	16.13	29.17	76.67	1	11.1		8	88.9	
	UMF 37 Mineral Angangeo	11	4	36.4		7	63.6	58.21	13.00	38.42	76.67	0	0.0		11	100.0	
	UMF 48 Huetamo	9	1	11.1		8	88.9	46.68	11.66	30.83	68.42	0	0.0		9	100.0	
	UMF 50 Maravatío	45	15	33.3		30	66.7	37.97	16.40	13.42	72.83	23	51.1		22	48.9	
	UMF 61 Tuzantla	3	0	0.0		3	100.0	45.89	15.29	28.33	56.25	1	33.3		2	66.7	
	UMF 79 Tlalpujahua	8	3	37.5		5	62.5	41.97	19.84	15.42	69.00	3	37.5		5	62.5	
	UMFH 18 Zitácuaro	17	10	58.8		7	41.2	52.08	19.23	21.58	85.33	3	17.6		14	82.4	
	UMFH 19 Cd. Hidalgo	179	84	46.9		95	53.1	38.27	16.94	8.92	82.58	76	42.5		103	57.5	
	Total	281	119	42.3		162	57.7	40.68	17.44	8.92	85.33	107	38.1		174	61.9	
Lázaro Cárdenas	HGZMF 12 Lázaro Cárdenas	26	16	61.5		10	38.5	56.68	13.10	23.92	76.42	2	7.7		24	92.3	
	UMF 23 Infiernillo	2	0	0.0		2	100.0	44.79	19.62	30.92	58.67	0	0.0		2	100.0	
	UMF 27 La Mira	13	5	38.5		8	61.5	58.98	11.80	27.17	73.58	1	7.7		12	92.3	
	UMF 31 Guacamayas	29	16	55.2		13	44.8	52.77	11.49	21.83	78.17	1	3.4		28	96.6	
	UMF 78 Lázaro Cárdenas	40	15	37.5		25	62.5	51.50	15.25	24.42	84.92	6	15.0		34	85.0	
	Total	110	52	47.3		58	52.7	53.82	13.57	21.83	84.92	10	9.1		100	90.9	
TOTAL		2471	977	39.5		1494	60.5	50.30	17.00	6.92	92.58	417	16.8		2064	83.2	

Table 4 presents the breakdown of demographic characteristics of patients for each primary healthcare clinic (UMF) of each subdelegation of description. Table 5 presents a summary of demographic characteristics of patients by Care unit.

**Table 5:** Demographic characteristics of all patients registered in the database at dialysis initiation for all causes (2007-2023), by Care unit (N=2471) HGR: Regional General Hospital; HGZ: Zone General Hospital; HGS: Subzone General Hospital; HGZMF: Zone General Hospital with Family Medicine; HGSMF: Subzone General Hospital with Family Medicine; UMAA: Ambulatory Care Medical Unit

Care Unit	Total, n	Sex						Age at dialysis initiation (years)				Age category at dialysis initiation					
		F			M			Mean	SD	Min	Max	Age ≤ 30 years			Age > 30 years		
		n		%	n	%						n		%	n	%	
HGR 1 Charo	687	284	41.3		403		58.7	47.01	17.93	8.92	87.83	167	24.2		524		75.8
HGS 7 La Piedad	94	33	35.1		61		64.9	49.34	16.29	17.17	83.08	18	19.1		76		80.9
HGSMF 17 Los Reyes	27	10	37.0		17		63.0	51.71	14.99	22.00	74.42	3	11.1		24		88.9
HGSMF 9 Apatzingán	63	31	49.2		32		50.8	51.15	16.92	13.08	79.33	8	12.7		55		87.3
HGZ 4 Zamora	241	79	32.8		162		67.2	52.12	16.39	7.08	84.50	34	14.0		208		86.0
HGZ 8 Uruapan	325	116	35.7		209		64.3	52.11	16.13	8.00	87.67	42	12.9		283		87.1
HGZ 83 Morelia	382	157	41.1		225		58.9	52.11	16.81	16.58	87.58	41	10.7		343		89.3
HGZMF 12 Lázaro Cárdenas	110	52	47.3		58		52.7	53.80	13.51	21.83	84.92	10	9.0		101		91.0
HGZMF 2 Zacapu	89	34	38.2		55		61.8	51.36	15.98	20.25	78.58	14	15.4		77		84.6
UMF 75 -UMAA 254 Morelia	453	181	40.0		272		60.0	50.45	17.22	6.92	92.58	80	17.7		373		82.3
TOTAL	2471	977	39.5		1494		60.5	50.30	17.00	6.92	92.58	417	16.8		2064		83.2

Provision of PD/HD services

**Figure 7:**
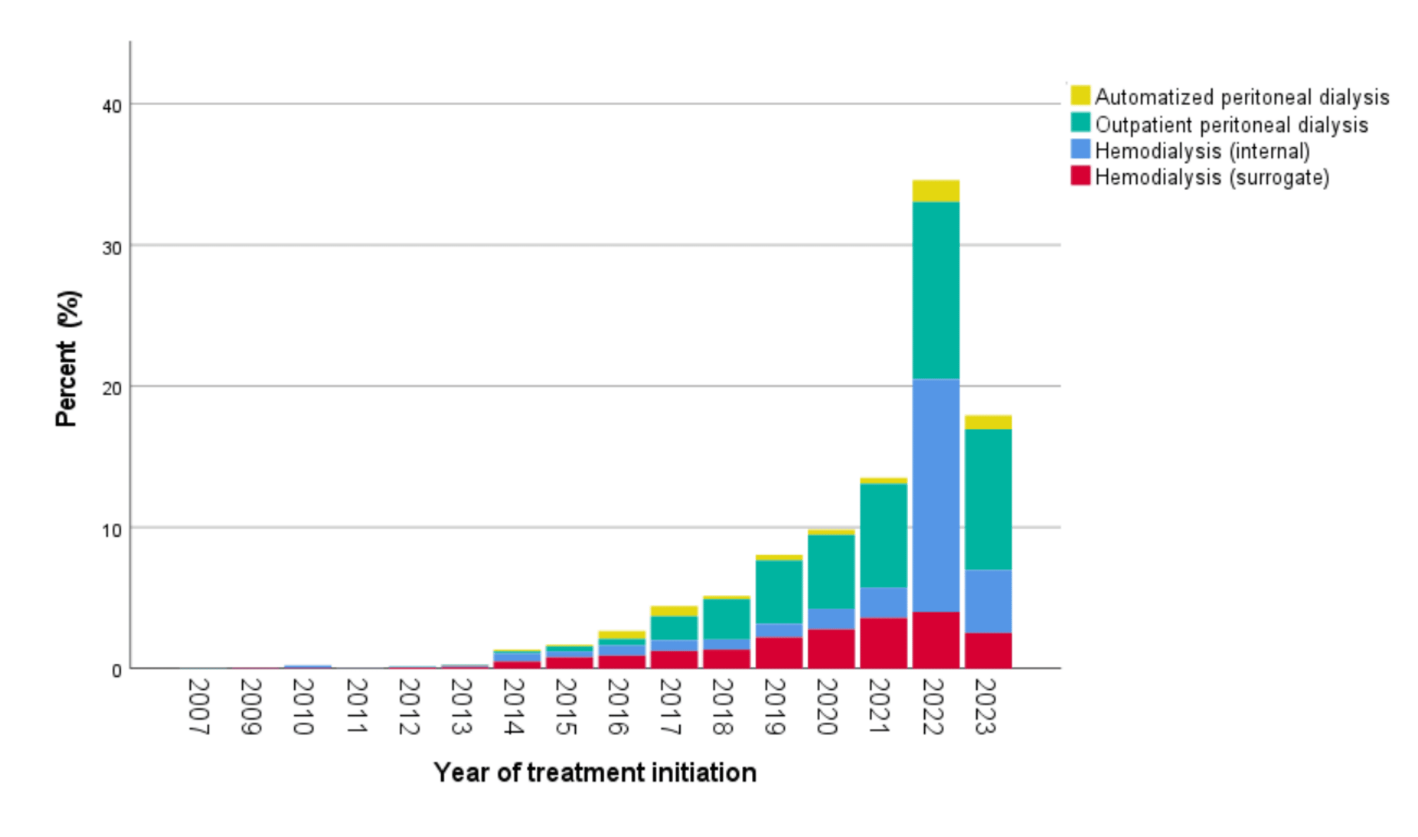
Evolution of the provision of dialysis services during the study period (2014-2023).

In Table 6 and Figure 7, the evolution of dialysis services at the moment of referral is shown.

**Table 6:** Evolution of the type of service provided to patients referred to dialysis (PD/HD) at the admission (2007-2023) ^a^ There were 45 missing values due to the absence of the year of admission to the service; ^b ^Internal HD refers to intramural HD (service provided at the IMSS facilities). PD: peritoneal dialysis; HD: hemodialysis; IMSS: Instituto Mexicano del Seguro Social

Service	2007-2015		2016		2017		2018		2019		2020		2021		2022		2023		TOTAL^a^	
	n	%	n	%	n	%	n	%	n	%	n	%	n	%	n	%	n	%	n	%
Automated Peritoneal Dialysis	7	7.7	15	22.4	18	16.5	6	4.7	10	4.9	10	3.9	11	3.2	32	3.7	20	4.8	129	5.2
Continuous Ambulatory Peritoneal Dialysis	16	17.6	12	17.9	42	38.5	73	57.0	114	55.3	135	52.9	191	55.7	310	35.9	223	53.3	1116	45.0
Internal Hemodialysis^b^	31	34.1	16	23.9	19	17.4	17	13.3	25	12.1	38	14.9	52	15.2	426	49.3	113	27.0	737	29.7
Subrogated Hemodialysis	37	40.7	24	35.8	30	27.5	32	25.0	57	27.7	72	28.2	89	25.9	96	11.1	62	14.8	499	20.1
TOTAL	91	100	67	100	109	100	128	100	206	100	255	100	343	100	864	100	418	100	2481	100.0

Table 7 presents the absolute and relative frequencies of service provision to patients included in the database at the time of their referral by the Care unit during the entire study period. Currently, there are 10 Care units available for PD and HD. The three units with the highest patient attendance are HGR 1 Charo (n=715, 28.3%), UMAA 75 Morelia (n=454, 18.0%), and HGZ 83 Morelia (n=384, 15.2%). Regarding the services provided within IMSS healthcare facilities, the most significant is continuous ambulatory peritoneal dialysis (n=1120, 44.3%), followed by internal hemodialysis (n=748, 29.6%) and automated peritoneal dialysis (n=130, 5.1%); the remaining HD services were outsourced (n=528, 20.9%). Analyzing the service provision in each Care unit, all of them provide continuous ambulatory peritoneal dialysis, but only six out of 10 units offer internal hemodialysis services (HGR 1, HGZ 4, HGZ 8, HGZ 83, HGZMF 2, and UMAA 75). Automated PD is only offered at UMAA 75, and outsourced hemodialysis has been implemented in four Care units (HGR 1, HGZ 4, HGZ 8, HGZMF 12). 

**Table 7:** Type of service provided to patients in dialysis treatment (PD/HD) for all causes (2007-2023), by Care unit and subdelegation of adscription, at the admission HGR: Regional General Hospital; HGZ: Zone General Hospital; HGS: Subzone General Hospital; HGZMF: Zone General Hospital with Family Medicine; HGSMF: Subzone General Hospital with Family Medicine; UMAA: Ambulatory Care Medical Unit

Service	Automated peritoneal dialysis		Continuous ambulatory peritoneal dialysis		Internal hemodialysis		Subcontracted hemodialysis		TOTAL	
	n	% (row)	n	% (row)	n	% (row)	n	% (row)	n	% (column)
by Care Unit										
HGR 1 Charo	0	0.0	404	56.5	153	21.4	158	22.1	715	28.3
HGS 7 La Piedad	0	0.0	94	100.0	0	0.0	0	0.0	94	3.7
HGSMF 17 Los Reyes	0	0.0	27	100.0	0	0.0	0	0.0	27	1.1
HGSMF 9 Apatzingán	0	0.0	63	100.0	0	0.0	0	0.0	63	2.5
HGZ 4 Zamora	0	0.0	80	31.4	64	25.1	111	43.5	255	10.1
HGZ 8 Uruapan	0	0.0	86	26.1	33	10.0	211	63.9	330	13.1
HGZ 83 Morelia	0	0.0	15	3.9	369	96.1	0	0.0	384	15.2
HGZMF 12 Lázaro Cárdenas	0	0.0	65	57.5	0	0.0	48	42.5	113	4.5
HGZMF 2 Zacapu	0	0.0	56	61.5	35	38.5	0	0.0	91	3.6
UMF 75 - UMAA 254 Morelia	130	28.6	230	50.7	94	20.7	0	0.0	454	18.0
by Subdelegation of Adscription										
Foreigners	1	7.1	3	21.4	4	28.6	6	42.9	14	0.6
Morelia	103	7.7	576	43.0	606	45.2	56	4.2	1341	53.1
Uruapan	0	0.0	148	37.9	34	8.7	209	53.5	391	15.5
Zamora	0	0.0	202	53.9	65	17.3	108	28.8	375	14.8
Zitácuaro	26	8.8	126	42.9	39	13.3	103	35.0	294	11.6
Lázaro Cárdenas	0	0.0	65	58.6	0	0.0	46	41.4	111	4.4
TOTAL	130	5.1	1120	44.3	748	29.6	528	20.9	2526	100.0

Table 7 presents the service provision by subdelegation of adscription also. Analyzing this information, only patients from two subdelegations (Morelia and Zitacuaro) had access to automated PD. Another interesting fact is that patients from subdelegations of Uruapan, Zamora, Zitácuaro, and Lazaro Cárdenas had minor proportions of patients referred to internal HD, compared to the subdelegation of Morelia, so they use subcontracted services.

**Figure 8:**
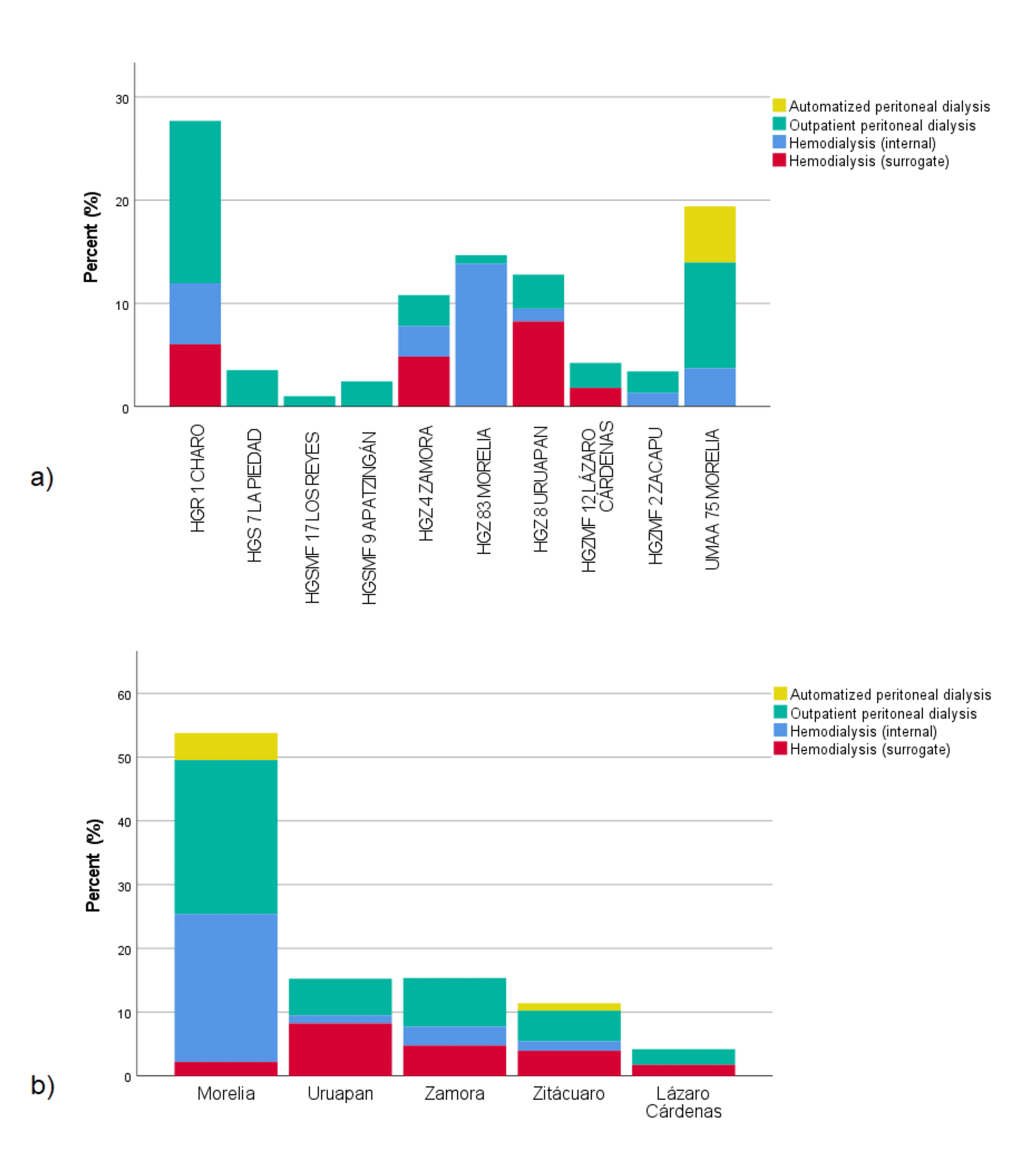
Provision of dialysis services, (a) by Care unit and (b) by subdelegation of adscription (2014-2023)

Figure 8 presents a graphical representation of the previously mentioned results.

## Discussion

Trends in RRT referral incidence

The database analysis allowed us to identify significant trends in the incidence of new cases of patients referred to RRT services at IMSS Michoacán. Generally, a growing trend has been observed since 2014, possibly due to various factors, including environmental aspects and epidemiological transitions. It should be mentioned that improved patient registration following the implementation of the database could be an important factor too. It is worth mentioning that this upward trend is also related to the increasing epidemiological trend of CKD that has been reported for the country in previous studies, including the analyses from the GBD project [14].

Additionally, the trend graphs (Figure 2) showed a more significant increase in patient referrals to RRT during the COVID-19 pandemic, suggesting a possible association between this disease and CKD progression. Notably, this increased incidence was also observed nationwide in a study that analyzed CKD incidence trends up to 2021 [14]. As in this study, the literature has reported an association between COVID-19 and CKD progression, leading to increased demand for RRT [28-30]. It is worth noting that the subdelegation with the most significant increase in new admissions was the Morelia subdelegation, where the Regional General Hospital 1 "Charo" is located, which is the most important medical referral center in the state of Michoacán, having the most extensive infrastructure.

Incidence rate of RRT referrals

For 2021, the incidence rate of patient admissions to RRT was estimated at 28.42 per 100,000 IMSS beneficiaries in Michoacán. In the same year, the GBD study estimated a CKD incidence rate for Michoacán at 436.1 per 100,000 population [6]. Assuming the same rate for IMSS beneficiaries in 2022, it would be determined that around 6.5% of new CKD-diagnosed patients progressed to RRT that same year. The incidence rate of admission to RRT was higher in men than women, which also aligns with the CKD incidence rate identified in previous studies, including the GBD. Regarding age, a growing trend in the incidence rate was observed as age groups increased, which seems reasonable, as the likelihood of requiring RRT increases with age, although this trend appears to decline.

Regarding the calculation of the incidence of admission to RRT by age group in the same year (2021), the high incidence in those under 20 years stands out, where there is a lower probability of being related to non-communicable diseases such as T2D and hypertension. Grouping the results obtained for those under 20 years of age, eight new cases were recorded, corresponding to an RRT admission incidence rate of 4.11 per 100,000 population. In the same year and for this age group, the GBD reported a CKD incidence in Michoacán of 53.25 per 100,000 population (15th place nationally) and a mortality rate of 1.39 per 100,000 population (6th place nationally) [6].

Regarding the calculation of the incidence of admission to RRT by the patient's subdelegation of adscription, the highest rate was for the Zitácuaro subdelegation (56.75 per 100,000 population), nearly double the rate obtained for the Morelia subdelegation (29.10 per 100,000 population). Additionally, the analysis conducted for the Eastern Michoacán region, which included the municipalities of Hidalgo, Zinapécuaro, and Maravatío, showed an incidence rate 6.46 times higher compared to the rest of the municipalities in the state of Michoacán. It is worth mentioning that these municipalities were grouped because they surround an area of interest, and the subdelegation analysis separates these municipalities as they belong to different subdelegations (the municipalities of Hidalgo and Maravatío belong to the Zitácuaro subdelegation, while the municipality of Zinapécuaro belongs to the Morelia subdelegation).

CKD causes recorded in the database

Regarding the results found concerning the causes of CKD at the time of patients' admission to RRT services, it stands out a high frequency of registrations as “Chronic Renal Insufficiency” or “Chronic Renal Insufficiency, not specified”. This relies on the electronic medical record (EMR) implemented, where medical staff can only enter one diagnosis instead of specifying a primary diagnosis, secondary diagnosis, and associations with other diseases. So, this registration could reflect various different etiologies, including non-communicable diseases and unknown causes (CKD of unknown etiology). The registration of particular etiologies showed non-communicable diseases such as diabetes and hypertension in both, those under 30 years of age and in those over 30 years of age; however, as expected, this was more significant in the latter group.

Age at dialysis initiation

One of this study's most important contributions is the analysis of the patients' age at dialysis initiation. The trend analysis for this variable (Figure 5) showed differences between patients' subdelegation of adscription and an increasing trend for this variable; this trend could be attributed to the epidemiological transition, which implies the impact of NCDs on renal impairment for the population. The distribution of this variable clearly showed a bimodal pattern (Figure 6), meaning it can be approximately divided into two different groups, with 40 years appearing to be the cut-off point. This analysis revealed no sex-related differences, but more importantly, this study found a difference concerning the subdelegation from which the patients originated, identifying Zitacuaro as the subdelegation where patients begin RRT at a younger age.

Furthermore, a special analysis comparing the age at dialysis initiation in patients from Care units located in the municipalities of Hidalgo, Zinapecuaro, and Maravatio with those from units in other municipalities showed that patients from Hidalgo, Zinapecuaro, and Maravatio had a noticeably younger age at dialysis initiation, averaging 12.44 years younger (Figure 5 and Figure 6).

RRT service provision

Another analysis described the type of RRT provided to patients during the analysis period. Table 4 and Figure 7 show the evolution in service provision, highlighting the increasing trend in hemodialysis service provision, even peaking in 2022, possibly related to the COVID-19 pandemic. This trend of increased hemodialysis provision has been documented across different institutions within the health system and may be due to better access to the necessary biomedical equipment. However, there is still much to be done to improve access to this service [31].

The comparative analysis of service provision by Care unit (Table 5 and Figure 8a) highlighted the lack of services in some units, such as automated PD, which is only provided at UMF 75 - UMAA 254 in Morelia, and internal (intramural) HD, which is not provided in four Care units (HGS 7 La Piedad, HGSMF 17 Los Reyes, HGSMF 9 Apatzingán, and HGZMF 12 Lázaro Cárdenas). Additionally, there is a high frequency of service outsourcing in four Care units (HGR 1 Charo, HGZ 4 Zamora, HGZ 8 Uruapan, and HGZMF 12 Lázaro Cárdenas). Furthermore, 46.3% (n=1169) of patients were treated in Care units located in the state capital (HGR 1 Charo and UMF 75 - UMAA 254 Morelia), highlighting the lack of infrastructure in the Care units in other municipalities of the state.

Analyzing RRT service provision by subdelegation of adscription (Table 5 and Figure 8b), the high frequency of services provided to the population affiliated with the Morelia subdelegation (53.1%) stands out, with a low frequency of outsourced services (4.2%). However, for the population affiliated with other subdelegations, low frequencies of HD services and very high frequencies of outsourced services (28.8-53.5%) were observed. This indicates the low availability of infrastructure for Hd in other subdelegations, which may pose a more significant burden on patients, as they must travel from their place of origin to receive this service.

Strengths and limitations of the study

The strengths of this study include the proposal to use the indicator of age at dialysis initiation, as this indicator could globally reflect the population's exposure to various risk factors, both individual (biological and behavioral) and environmental, occupational, and socioeconomic, which can jointly influence CKD development. Additionally, the analysis of the geographical location of municipalities and subdelegations of the institution helped identify the IMSS health service delivery system.

The study's limitations include that it was based solely on a retrospective analysis based on the database provided by IMSS's RRT services, which limits it to the information available in this database, which the implemented EMR system may constrain. Also, a time series analysis could have been a better analysis to have a statistical model that could generate predictive information, but we consider this could be a future analytic approach.

Public health implications

This study has several implications. From an epidemiological perspective, further study is needed to explore the risk factors associated with CKD incidence and referral to RRT. While studies have explored metabolic risk factors [22], the impact of environmental, socioeconomic, and occupational factors remains underexplored. Notably, other studies in Mexico have found an association between agricultural work and CKD incidence [17], making this an important area of research in Michoacán. Exposure to metals and chemicals also presents a significant research opportunity. Additionally, there is a need to establish an observatory to analyze this issue on a regional level in Michoacán, particularly in the eastern region of the state, assessing CKD prevalence and incidence from various causes, including those with unknown etiology.

From a biomedical perspective, expanding research on the causes of CKD incidence in the area of interest is crucial. This may require obtaining percutaneous renal biopsies from native kidneys, as done in another state [15]. Furthermore, incorporating genomic studies of affected patients is essential to identify potential gene associations with CKD incidence.

From a health systems perspective, it is essential to strengthen the infrastructure for HD within the institution to minimize the need for outsourced services, which have been documented to incur higher costs [32]. A holistic approach to patient management in PD is necessary, emphasizing enhancing kidney transplantation, which remains the most cost-effective and quality-of-life-improving therapy in the long term. Moreover, the lack of infrastructure can impose additional burdens on patients, requiring them to travel from their place of origin, incur transportation costs, and spend time commuting, thereby increasing the burden on primary caregivers. Also, regarding the previously mentioned problem in the EMR, this presents an opportunity to improve this patient registration and tracking system.

## Conclusions

The analysis conducted in this study identifies that there is an increasing trend in RRT referrals at the IMSS Michoacán that could be associated with the epidemiological transition phenomenon in Mexico. A more important issue in the incidence of RRT referrals and age at treatment initiation was identified for a particular subdelegation of adscription and region of interest in this institution. Moreover, the need to strengthen HD service infrastructure is identified to improve accessibility for insured patients living in various municipalities in Michoacán, Mexico. Future research should investigate environmental, behavioral, and occupational risk factors, especially in the region of interest, to understand the earlier onset of CKD.

## References

[1] Romagnani P, Remuzzi G, Glassock R (2017). Chronic kidney disease. Nat Rev Dis Primers.

[2] Martínez-Castelao A, Górriz JL, Segura-de la Morena J (2014). Consensus document for the detection and management of chronic kidney disease. Nefrologia.

[3] (2024). KDIGO 2024 clinical practice guideline for the evaluation and management of chronic kidney disease. Kidney Int.

[4] Ravender R, Roumelioti ME, Schmidt DW, Unruh ML, Argyropoulos C (2024). Chronic kidney disease in the older adult patient with diabetes. J Clin Med.

[5] Rosas-Valdez FU, Aguirre-Vázquez AF, Agudelo-Botero M (2024). Quantification of the burden of chronic kidney disease in Latin America: an invisible epidemic [Article in Spanish]. Rev Panam Salud Publica.

[6] (2024). Institute for Health Metrics and Evaluation (IHME): GBD compare. https://vizhub.healthdata.org/gbd-compare/.

[7] Lou-Meda R, Alvarez-Elías AC, Bonilla-Félix M (2022). Mesoamerican endemic nephropathy (MeN): a disease reported in adults that may start since childhood?. Semin Nephrol.

[8] Sanchez Polo V, Garcia-Trabanino R, Rodriguez G, Madero M (2020). Mesoamerican nephropathy (MeN): what we know so far. Int J Nephrol Renovasc Dis.

[9] Correa-Rotter R, García-Trabanino R (2019). Mesoamerican nephropathy. Semin Nephrol.

[10] Correa-Rotter R, Wesseling C, Johnson RJ (2014). CKD of unknown origin in Central America: the case for a Mesoamerican nephropathy. Am J Kidney Dis.

[11] Sánchez-Cedillo A, Cruz-Santiago José, Mariño-Rojas FB, Hernández-Estrada S, García-Ramírez C (2020). Burden of disease: end stage renal disease, dialysis-hemodialysis and kidney transplantation in Mexico [Article in Spanish]. Revista Mexicana de Trasplantes.

[12] Polanco-Flores NA (2019). Chronic renal disease in Mexico: A preventive uncontrolled epidemic. Rev Medica del Hosp Gen Mex.

[13] Chávez-Gómez NL, Cabello-López A, Gopar-Nieto R (2017). Chronic kidney disease in Mexico and its relationship with heavy metals [Article in Spanish]. Rev Med Inst Mex Seguro Soc.

[14] Argaiz ER, Morales-Juárez L, Razo C, Ong L, Rafferty Q, Rincón-Pedrero R, Gamba G (2023). The burden of chronic kidney disease in Mexico: data analysis based on the Global Burden of Disease 2021 study. Gac Med Mex.

[15] Gutierrez-Peña M, Zuñiga-Macias L, Marin-Garcia R (2021). High prevalence of end-stage renal disease of unknown origin in Aguascalientes Mexico: role of the registry of chronic kidney disease and renal biopsy in its approach and future directions. Clin Kidney J.

[16] Rosa-Diez G, Gonzalez-Bedat M, Ferreiro A, García-García G, Fernandez-Cean J, Douthat W (2016). Burden of end-stage renal disease (ESRD) in Latin America. Clin Nephrol.

[17] Aguilar-Ramirez D, Raña-Custodio A, Villa A (2021). Decreased kidney function and agricultural work: a cross-sectional study in middle-aged adults from Tierra Blanca, Mexico. Nephrol Dial Transplant.

[18] Villalvazo P, Carriazo S, Martin-Cleary C, Ortiz A (2021). Aguascalientes: one of the hottest chronic kidney disease (CKD) hotspots in Mexico and a CKD of unknown aetiology mystery to be solved. Clin Kidney J.

[19] Lozano-Kasten F, Sierra-Diaz E, de Jesus Celis-de la Rosa A, Margarita Soto Gutiérrez M, Aarón Peregrina Lucano A (2017). Prevalence of albuminuria in children living in a rural agricultural and fishing subsistence community in Lake Chapala, Mexico. Int J Environ Res Public Health.

[20] Cárdenas-González M, Osorio-Yáñez C, Gaspar-Ramírez O (2016). Environmental exposure to arsenic and chromium in children is associated with kidney injury molecule-1. Environ Res.

[21] Esparza-Aguilar M, Ochoa-Esquivel R del C, Barajas-González A, Ávila-Rosas H (2019). Mortality in Mexico due to chronic kidney disease in children under 20 years of age, 2000-2014. Rev Mex Pediatría.

[22] Alvarez Paredes AR, Gómez García A, Alvarez Paredes MA (2024). Prevalence and metabolic risk factors of chronic kidney disease among a Mexican adult population: a cross-sectional study in primary healthcare medical units. PeerJ.

[23] (2024). Instituto Nacional de Estadística y Geografía (INEGI): Population and housing census 2020 [Webpage in Spanish]. https://www.inegi.org.mx/programas/ccpv/2020/.

[24] (2024). Instituto Mexicano del Seguro Social (IMSS): Catálogo de Unidades de Medicina Familiar de primer nivel de la
Dirección de Prestaciones Médicas del Instituto Mexicano del Seguro Social. Catalogue of First Level Family Medicine Units of the Medical Benefits Directorate of the Mexican Social Security Institute [Document in Spanish].

[25] Instituto Mexicano del Seguro Social (IMSS (2024). Instituto Mexicano del Seguro Social (IMSS): Catalog of IMSS facilities [Website in Spanish]. https://www.imss.gob.mx/directorio/?page=1.

[26] González Block MA, Morales HR, Hurtado LC, Balandrán A, Méndez E (2024). Ministry of Health: Catalog of unique health establishment codes (CLUES) 2023. Health Systems in Transition: Mexico Health System Review 2020.

[27] (2024). QGIS: Spatial without compromise. https://qgis.org/es/site/.

[28] Geetha D, Kronbichler A, Rutter M (2022). Impact of the COVID-19 pandemic on the kidney community: lessons learned and future directions. Nat Rev Nephrol.

[29] Chávez-Valencia V, Orizaga-de-la-Cruz C, Lagunas-Rangel FA (2022). Acute kidney injury in COVID-19 patients: pathogenesis, clinical characteristics, therapy, and mortality. Diseases.

[30] Kant S, Menez SP, Hanouneh M (2020). The COVID-19 nephrology compendium: AKI, CKD, ESKD and transplantation. BMC Nephrol.

[31] García-García G, García-Bejarano H, Breien-Coronado H (2017). End-stage renal disease in Mexico. Chronic Kidney Disease in Disadvantaged Populations.

[32] Méndez Durán A, Ignorosa Luna M, Pérez Aguilar G, River Rodrigueza F, González Izquierdo JJ, Dávila Torres J (2016). Current status of alternative therapies renal function at the Instituto Mexicano del Seguro Social [Article in Spanish]. Rev Med Inst Mex Seguro Soc.

